# Diagnosis and treatment of communicating bronchopulmonary foregut malformation: Report of two cases and review of the literature

**DOI:** 10.1097/MD.0000000000006307

**Published:** 2017-03-24

**Authors:** Hongxia Ren, Liqiong Duan, Baohong Zhao, Xiaoxia Wu, Hongyi Zhang, Caixia Liu

**Affiliations:** Department of Pediatric Surgery, Children's Hospital of Shanxi Province, Shanxi, China.

**Keywords:** case report, communicating bronchopulmonary foregut malformation, esophageal atresia, neonate

## Abstract

**Rationale:**

Communicating bronchopulmonary foregut malformation (CBPFM) is a rare congenital malformation involving both the digestive and respiratory systems. To our best knowledge, most cases of CBPFM reported in the literature were in infancy or adulthood and CBPFM in infantile is even rarer with a high case-fatality rate partly due to misdiagnosis.

**Patient concerns:**

We presented 2 cases of neonatal CBPFM. A 11-hour male newborn was admitted because of moaning for 7 hours, and a 1-day male newborn was referred to us with profuse foams, choking on breast-milk feeding and facial cyanosis.

**Diagnoses:**

With the assistance of upper gastrointestinal tract imaging and contrast-enhanced chest computed tomography (CT), the diagnosis was established according to the most recent diagnostic criteria.

**Interventions:**

The case one recieved a lower left pneumonectomy and surgical repair of esophageal fistula. The case two was performed with a surgical repair of esophageal atresia and esophageal tracheal fistula firstly, and then also received a repair of communicating bronchopulmonary foregut malformation two weeks after the first operation.

**Outcomes:**

The case one was cured and discharged 2 weeks after admission. Unfortunately the case two died from respiratory failure.

**Lessons:**

Pediatric surgeons should therefore be aware that type I CBPMF is rare and preoperative diagnosis is usually difficult. Maldiagnosis is uncommon because clinicians often focus their attention on esophageal atresia and neglect pulmonary abnormalities. Other than upper gastrointestinal tract radiography and CT scan, bronchoscopy should be considered in pediatric patients with esophageal atresia complicated with pulmonary abnormalities, knowing that bronchoscopy may help confirm the diagnosis and select surgical strategies.

## 1 Introduction

Communicating bronchopulmonary foregut malformation (CBPFM) is a rare congenital malformation involving both the digestive and respiratory systems characterized by congenital pulmonary sequestration and the formation of an abnormal passage in the digestive tract (esophagus or stomach).^[1]^ Most cases of CBPFM reported in the literature occurred in infancy or adulthood, and rarely in the neonatal period. This article reports 2 cases of CBPFM in neonates admitted in our hospital. Their clinical symptoms and signs and the main points of treatment were summarized retrospectively, and the related literature was reviewed in an attempt to enhance the understanding about this rare disease.

## 2 Ethic statement

This ethical approval was not necessary for this study. We have consulted the ethics committee of the Children's Hospital of Shanxi Province and they stated that ethical approval was not necessary for our study. Written informed consents were obtained from the patients.

## 3 Cases report

### 3.1 Case 1

**Figure 1:**
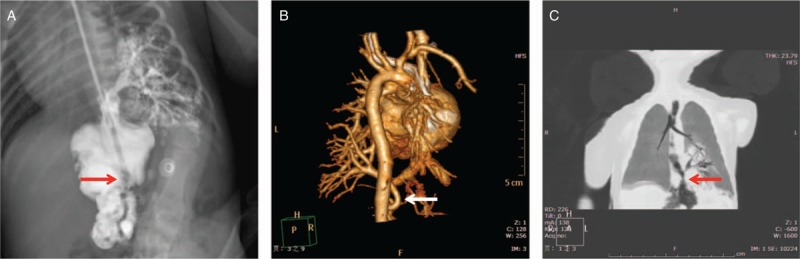
Picture from Case 1. A, Upper gastrointestinal tract radiography (arrow indicating an abnormal esophagobronchial passage). B, CT 3D reconstruction (arrow indicating an abnormal blood supply). C, CT esophagobronchial 3D reconstruction (arrow indicating an abnormal esophagobronchial passage). 3D = three-dimensional, CT = computed tomography.

A G1P1 male newborn was admitted 11 hours after Cesarean section at 40 gestational weeks because of moaning for 7 hours. Feeding intolerance, vomiting, and abdominal distension were noticed after admission. Physical examination revealed poor response, weak crying, cyanosis of the mouth, lips, face and 4 extremities, nasal flaring, positive 3-depression sign, and 78% percutaneous oxygen saturation. Digestive tract imaging after admission (Fig. 1A) revealed congenital bronchopulmonary foregut malformation and volvulus of the stomach; chest CT three-dimensional (3D) vascular reconstruction (Fig. 1B) suggested abnormal blood supply originating from systemic circulation; chest CT 3D esophagotracheal reconstruction (Fig. 1C) suggested soft tissue density shadows in the posterior basal segment of the left lower lung lobe, in which clusters of spotty high-density bronchial shadows were seen, and the bronchial shadows in the affected area were patent with the esophagus. Echocardiography showed (central type) atrial septal defect (ASD) and patent ductus arterious (PDA). A diagnosis of “CBPFM, ASD, and PDA” was made. At day 3 after admission, surgery was performed, confirming the diagnosis of introlobar pulmonary sequestration in the left lower lung lobe. The left lower lung lobe was seen adhering with the surrounding chest wall. Multiple small vessels communicating with the intercostals vessels and thoracic aorta were sharply dissected, ligated, and resected. The intraesophageal fistula was cut and sutured. Tube-feeding was initiated 5 days after surgery, and oral feeding was initiated 10 days after surgery. Upper gastrointestinal tract imaging showed no contrast medium leakage. The patient was cured and discharged 2 weeks after admission. Pathology reported that the submitted (left lower lung) tissue was consistent with pulmonary sequestration, in which large amounts of inflammatory cell infiltration and alveolar and bronchial epithelial proliferation were noticed. Following up half a year, the child grew up normally without any discomfort.

### 3.2 Case 2

**Figure 2:**
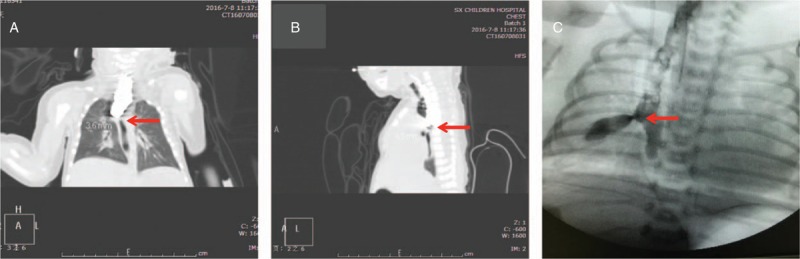
Picture from Case 2. A, Esophageal radiography (arrow indicating the esophageal blind end). B, CT sagittal plane (arrow indicating the distance between the 2 esophageal blind ends). C, Esophageal radiography 2 weeks after esophageal anastomosis (arrow indicating an abnormal esophagobronchial passage). CT = computed tomography.

A 1-day-old G_1_P_1_ full-term naturally delivered male newborn was admitted to our hospital because of profuse foams, choking on breast-milk feeding, and facial cyanosis. Upper gastrointestinal tract imaging revealed esophageal atresia (type III); echocardiography revealed acleistocardia; chest CT revealed tracheal atresia (Fig. 2A); the lateral view showed the distance between the 2 blind ends of the esophagus as 0.45 cm (Fig. 2B). A diagnosis of “type III esophageal atresia and acleistocardia” was made. Thoracoscopic exploration and esophageal anastomosis under general anesthesia were scheduled. Aerothorax was established after anesthesia. However, the child was unable to tolerate mechanic ventilation due to unknown reasons. Seeing that his blood oxygen began deceasing and vital signs became unstable, the procedure was converted to open surgery for 1-stage esophageal anastomosis and fistula repair. The distance between the 2 esophageal blind ends was found to be 0.8 cm. The operation was smooth. Two days after operation, the endotracheal intubation was removed, but the child still had dyspnea, which was considered due to laryngeal edema, for which dexamethasone (2 mg, bid, for 2 days) and budesonide (2 mL, tid, for 7 days) were administered by atomizing inhalation. Two weeks after operation, the symptom of dyspnea became worse, for which endotracheal intubation and respirator-assisted respiration were implemented again. During this period, the patient's abdominal distention became worse and large amounts of gas were seen coming into the nasogastric decompression aspirator, which was considered due to an esophagotracheal fistula. Contrast-enhanced C-arm esophagography showed that the original esophageal anastomosis healed well, and there was an abnormal passage in the esophagus at T6 level (Fig. 2C). A diagnosis of (type I) CBPFM with esophageal atresia was suspected. An emergency operation was performed again by dissecting the esophagus, resecting the abnormal tissue between the esophagus and trachea, and sewing up the esophagus. However, the respiratory failure was not improved after the operation, and the child died the following day.

## 4 Discussion

In 1968, Gerle et al^[2]^ first put forward the diagnosis of bronchopulmonary foregut malformation (BPFM), which was referred to as the abnormal passage between the bronchus and the stomach or the esophagus on the basis of congenital pulmonary sequestration. Srikanth et al^[3]^ classify CBPFM into 4 types: type I refers to esophageal atresia, where there is a passage between the distal end of atresia and the sequestrated lung; type II refers to the presence of an undeveloped lung with a mass in the unilateral chest cavity that originates from the lower segment of the esophagus; type II refers to communication of the sequestrated lung lobe or segment with the esophagus or stomach; type IV refers to communication of part of the normal bronchial system with the esophagus. According to reports in the literature, type III CBPFM is the most common, followed by type II. There have been only few reports about type I CBPFM. In addition, both the misdiagnosis rate and mortality rate of type I CBPFM are high. In the 2 cases reported herein, Case 1 belongs to type III, and Case 2 belongs to type I.

CBPFM may occur at any time from infancy to adulthood, but most cases were detected several months after birth. The male/female ratio of CBPFM is about 1:2. The most common symptoms are chronic cough that becomes worse on feeding, recurrent pneumonia, hemoptysis, vomiting, and respiratory distress.^[4]^ CBPFM is often accompanied with other malformations such as Goldenhar syndrome, congenital heart disease, and spinal distortion. The patient in Case 1 of this series was accompanied with ASD and PDA. The imaging characteristics of CBPFM include pulmonary sequestration and an abnormal open passage with the gastrointestinal tract. Upper gastrointestinal tract imaging can display the position of the abnormal passage between the digestive tract and the trachea, and the presence or absence of gastroesophageal reflux and volvulus of the stomach. It can also display the position of the blind-end of esophageal atresia in type I CBPFM. CT scan and 3D reconstruction can display lung parenchymal lesions, intra- or extra-lobar type, abnormal blood supply of the sequestrated lung, and other developmental abnormalities and associated malformations.^[5]^ Upper gastrointestinal tract imaging and contrast-enhanced chest CT have become the optimal selection in the diagnosis of CBPFM.

In the present report, the diagnosis of Case 1 was confirmed by upper gastrointestinal tract imaging and contrast-enhanced chest CT, and the patient was clinically cured soon after surgical intervention. However, upper gastrointestinal tract imaging and contrast-enhanced chest CT in Case 2 failed to demonstrate the intrapulmonary lesions, because our attention was fully focused on the diagnosis of esophageal atresia at the moment. Although unexplained postoperative clinical manifestations were present, no timely examination was performed until 2 weeks later, when the diagnosis of CBPMF was confirmed by a second contrast-enhanced C-arm esophagography. No genetic tests were performed in the 2 cases. Our literature research shows that several individual case reports about the diagnosis and treatment of the disease have been published in recent years,^[6–8]^ in which neonatal CBPMF was initially diagnosed as esophageal atresia without exception, and the diagnosis was confirmed only when CT and esophagoscopy were performed again because of persistent dyspnea after the operation. Pediatric surgeons should therefore be aware that type I CBPMF is rare and preoperative diagnosis is usually difficult. Maldiagnosis is uncommon because clinicians often focus their attention on esophageal atresia and neglect pulmonary abnormalities. In their retrospective study on preoperative bronchoscopy in 62 newborns who were diagnosed with esophageal atresia, Atzori et al^[9]^ found that bronchoscopy could help ascertain the type of esophageal atresia, and find esophagotracheal abnormalities. We therefore suggest that other than upper gastrointestinal tract radiography and CT scan, bronchoscopy should be considered in pediatric patients with esophageal atresia complicated with pulmonary abnormalities, knowing that bronchoscopy may help confirm the diagnosis and select surgical strategies.

Surgery remains the only treatment for neonatal CBPFM at present, and thoracoscopic therapy for CBPFM has also been reported in most recent years with good postoperative recovery.^[10–12]^ In Case 1 of this report, the diagnosis was confirmed preoperatively and left lower lung resection was performed smoothly and the patient recovered uneventfully after operation. A recent follow-up visit shows that the child is growing well. In Case 2, we failed to detect pulmonary malformation before esophageal anastomosis and the patient presented persistent respiratory distress. Although surgery was performed again after confirmation of the diagnosis, respiratory failure was not improved despite a second operation, and the patient died in the end, which deserves clinical concern and attention.

5. Summary Upper gastrointestinal tract imaging and contrast-enhanced chest CT have become the optimal selection in the diagnosis of CBPFM.CBPFM in neonatal is rare and those cases with an early precise diagnosis could get an uneventful recovery.
